# Efficacy and Safety of Different Norepinephrine Regimens for Prevention of Spinal Hypotension in Cesarean Section: A Randomized Trial

**DOI:** 10.1155/2018/2708175

**Published:** 2018-05-23

**Authors:** Daili Chen, Xiaofei Qi, Xiaolei Huang, Yang Xu, Feilong Qiu, Yuting Yan, Yuantao Li

**Affiliations:** Department of Anesthesiology, Shenzhen Maternity and Child Healthcare Hospital, Southern Medical University, Shenzhen, Guangdong 518028, China

## Abstract

The aim of this paper is to evaluate the efficacy and safety of three different norepinephrine dosing regimens for preventing spinal hypotension in cesarean section. In this randomized double-blinded controlled study, 120 parturients scheduled for elective section delivery under spinal anesthesia were assigned to 1 of 4 groups. In the control group, patients received saline infusion. In three norepinephrine groups, the infusion dosage regimens were 5, 10, and 15 *μ*g/kg/h, respectively. Hypotension was treated with a rescue bolus of 10 *μ*g norepinephrine. The study protocol was continued until the end of surgery. The primary outcome was the proportion of participants that underwent hypotension. The proportion of hypotension participants was significantly reduced in the norepinephrine groups (37.9%, 20%, and 25%, respectively) compared to that in the control group (86.7%). However, the highest dose of norepinephrine (15 *μ*g/kg/h) resulted in more hypertension episodes. In addition, blood pressure was better maintained in the norepinephrine 5 *μ*g/kg/h and 10 *μ*g/kg/h groups than in the control group and 15 *μ*g/kg/h group. No significant differences in other hemodynamic variables, adverse effects, maternal and neonatal blood gases, or Apgar scores were observed among the groups. In summary, for patients who undergo cesarean delivery under spinal anesthesia, infusion of 5–10 *μ*g/kg/h norepinephrine was effective to reduce hypotension incidence without significant adverse effects on maternal and neonatal outcomes. Clinical Trial Registration Number is ChiCTR-INR-16009452.

## 1. Introduction 

During spinal anesthesia for cesarean delivery, maternal hypotension is a major complication with the incidence up to 60–70% [1]. Prolonged hypotension leads to decreased uteroplacental blood flow [2, 3] and fetal acidosis [4]. Vasopressors such as ephedrine, phenylephrine, and norepinephrine are therefore commonly recommended to reduce the incidence of hypotension. Compared with ephedrine, phenylephrine seems to be a better choice due to a lower incidence of fetal acidosis and maternal nausea and vomiting [5, 6]. However, the use of phenylephrine as the first-line agent may lead to bradycardia and reduce cardiac output [7–9].

A recent study indicated that norepinephrine infusion during spinal anesthesia for cesarean delivery was associated with greater heart rate and cardiac output compared with phenylephrine [10]. Other studies also showed that norepinephrine could act as an alternative to phenylephrine without adverse outcomes [11, 12]. However, the optimal regimen for norepinephrine infusion has not been determined.

In this study, we aimed to assess the efficacy and safety of three different norepinephrine regimens for preventing hypotension in cesarean section under spinal anesthesia and determine the optimal regimen for clinical practice. The primary outcome was the proportion of hypotension participants. We hypothesized that the proportion of hypotension would be reduced and that hemodynamics would be better maintained with the use of norepinephrine.

## 2. Materials and Methods

### 2.1. Patients and Medications

This randomized double-blinded controlled study was conducted in the Shenzhen Maternity and Child Healthcare Hospital, China. Ethical approval was obtained from the Ethical Committee (reference no. SZFY2016080101, Chairperson Prof. JL. Yao). This study was registered in the Chinese Clinical Registry Center (registration no. ChiCTR-INR-16009452). All participants gave their written informed consent.

Inclusive criteria included American Society of Anesthesiologists physical status 1 or 2, age 18–40 years, full-term pregnancy, and scheduled for elective cesarean section under spinal anesthesia. Exclusion criteria included cardiac diseases, hypertension, diabetes, recent use of vasoactive medications, arrhythmia, any contraindication to spinal anesthesia, and known fetal abnormality or fetal distress.

Patients were assigned to 1 of 4 groups according to a computer-generated randomization sequence: a control group and three norepinephrine groups (NE 1, NE 2, and NE 3). To ensure allocation concealment, each randomization code was placed in a sealed, opaque, consecutively numbered envelope by an independent medical staff. An anesthesia resident who was not involved with the conduct of the study prepared the medications in the syringes according to the randomization codes. In the control group, the infusion syringe contained 50 ml of normal saline. In the three norepinephrine groups, the infusion syringe contained 50 ml norepinephrine (GrandPharmr. Co. Ltd., H42021301, Wuhan, China) diluted with saline to concentrations of 5, 10, and 15 *μ*g/ml, respectively. For all the groups, the bolus syringe contained norepinephrine at 10 *μ*g/ml diluted with saline. The investigator who administered the study medications and collected the data was unaware of the group assignment.

### 2.2. Protocol

Patients were fasted overnight, and no premedication was administered. In the operating room, standard monitoring included electrocardiography, pulse oximetry, and noninvasive hemodynamic monitoring with LIDCO rapid (HM81-01, LiDCO Ltd., London, United Kingdom). Baseline systolic blood pressure (SBP), mean blood pressure (MBP), diastolic blood pressure (DBP), heart rate (HR), cardiac output (CO), and vascular resistance (SVR) were recorded. All measurements were continuously recorded until the end of surgery. An upper limb vein was cannulated with a 20 G intravenous catheter. To avoid possible influence on the measurements, the monitoring module was placed on the other arm. Lactated Ringer's solution of 10 ml/kg was administered within 20 min, followed by a maintenance rate of 20 ml/min.

Patients were placed in the right lateral position. Spinal anesthesia was performed with a 25 G pencil point needle at the L3–4 interspace. After confirming the cerebrospinal fluid, 2.2–2.5 ml of 0.5% ropivacaine plus 0.1 mg morphine was administered intrathecally at the rate of 0.1 ml/s. Then the patients were placed in the tilted supine position. Oxygen was delivered via mask at 5 L/min. Block level was assessed by pinprick with a 23 G needle and controlled within T4–6. If the anesthesia level was higher than T4, the case was excluded from analysis. If blood loss exceeded 500 ml, the case was also excluded from analysis.

Study procedures are shown in Figure S1. Immediately after intrathecal injection, the study medications were started at 1 ml/kg/h using an infusion pump. In the three norepinephrine groups, the infusion dosage regimens were 5, 10, and 15 *μ*g/kg/h, respectively. In the control group, patients received saline infusion. A rescue bolus of 10 *μ*g norepinephrine from the bolus syringe was used to treat hypotension. The study protocol was continued until the end of surgery. After delivery, intravenous oxytocin of 5 U was administered slowly and another 5 U was injected into the uterine muscle.

For our study, hypotension was defined as SBP < 80% of the baseline value or < 90 mmHg. Hypertension was defined as a 20% increase over the baseline BP value. When hypertension occurred, the infusion was stopped. Bradycardia (heart rate < 50 beats/min) was treated with 0.3 mg atropine.

Hemodynamic values including SBP, MBP, DBP, HR, CO, and SVR were recorded at five timepoints: baseline (T1), block of the highest sensory level (T2), delivery (T3), oxytocin administration (T4), and end of surgery (T5). Norepinephrine consumption before delivery and during the surgery was recorded. Adverse effects including shivering, vomiting, peripheral vascular constriction, bradycardia, hypertension, and hypotension were recorded. In addition, maternal venous blood gases and neonatal outcomes including the Apgar scores at 1 and 5 min and umbilical venous blood gases were assessed.

### 2.3. Primary and Secondary Outcomes

The primary outcome of this study was the proportion of hypotension participants. The proportion was calculated by percentage of patients who experienced hypotension no matter how many episodes she experienced. The secondary outcomes included hemodynamic changes, norepinephrine consumption, adverse effects, maternal and neonatal blood gas, and Apgar scores.

### 2.4. Statistical Analysis

Sample size was calculated with PASS 11 software. According to a pilot study with a hypotension incidence of 76%, a sample size of 28 patients per group would be required to detect a difference of 20% with two-sided *α* error of 0.05 and 80% power. To compensate for possible dropouts or excluded cases, we included 30 patients in each group.

Statistical analysis was conducted with SPSS 20.0 software (IBM SPSS, Chicago, Illinois). Data were tested for normality using the Kolmogorov–Smirnov test. Continuous variables are presented as mean ± standard deviation (SD), and categorical variables are presented as number (*n*, %). Continuous variables were analyzed by analysis of variance (ANOVA) with post hoc multiple comparisons by Student–Newman–Keuls- (SNK-) *q* test. For categorical variables, the Chi-square (*χ*^2^) test or Fisher's exact test was used as appropriate, and the partition of *χ*^2^ method was used for multiple comparisons. Multiple comparisons between multiple sample rates were used partitions of *χ*^2^ method, *α*′ = *α*/[*k*(*k* − 1)/2 + 1]. In our study there were 4 groups, so *k* = 4, *α*′ = 0.05/7 = 0.00714. Two-tailed *p* values of 0.05 were considered statistically significant.

Hemodynamic data at each individual time point were compared by ANOVA. Differences within subjects, differences between subjects, and interactions of two factors were compared with repeated measurements by SNK-*q* test.

## 3. Results

**Figure 1:**
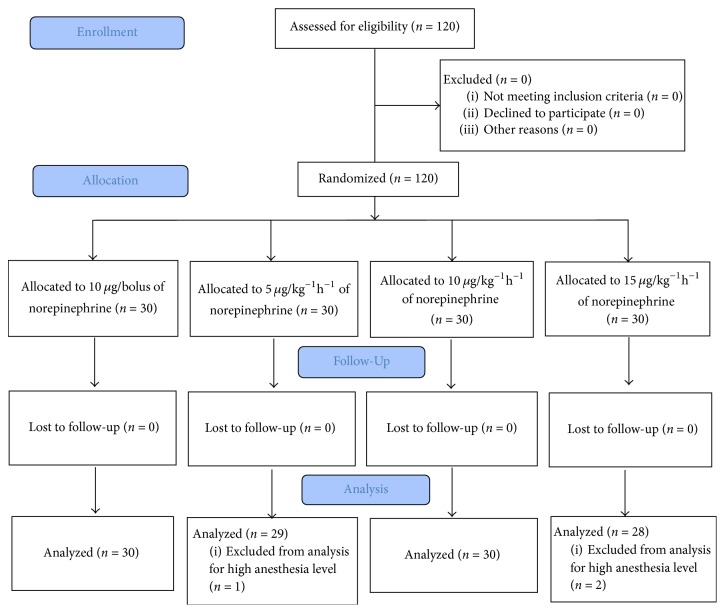
CONSORT flow chart.

The study was conducted between November 3, 2016, and March 17, 2017. A total of 120 parturients were enrolled into the trial. Most patients (112 in 120 patients) were with a successful spinal anesthesia at the first attempt except 8 patients who received twice puncture attempt. Nobody experienced blood loss more than 500 ml. Three patients were excluded due to high block level and 117 participants were enrolled to final analysis. The CONSORT diagram is shown in Figure 1. Patient demographic and surgical characteristics are shown in Table 1. Norepinephrine consumption was shown in Table 2.

### 3.1. Hypotension and Hypertension

The proportion of participants who experienced hypotension and hypertension are shown in Table 3. The proportion of hypotension was significantly lower in the all norepinephrine groups (86.7% in control group, 37.9% in NE 1, 20% in NE 2, and 25% in NE3, *p* < 0.001). However, patients who received the highest dose of norepinephrine (15 *μ*g/kg/h) experienced more hypertension episodes than those who received 5 or 10 *μ*g/kg/h norepinephrine (75% versus 41.4% and 36.6%, respectively).

### 3.2. Hemodynamic Changes

**Figure 2:**
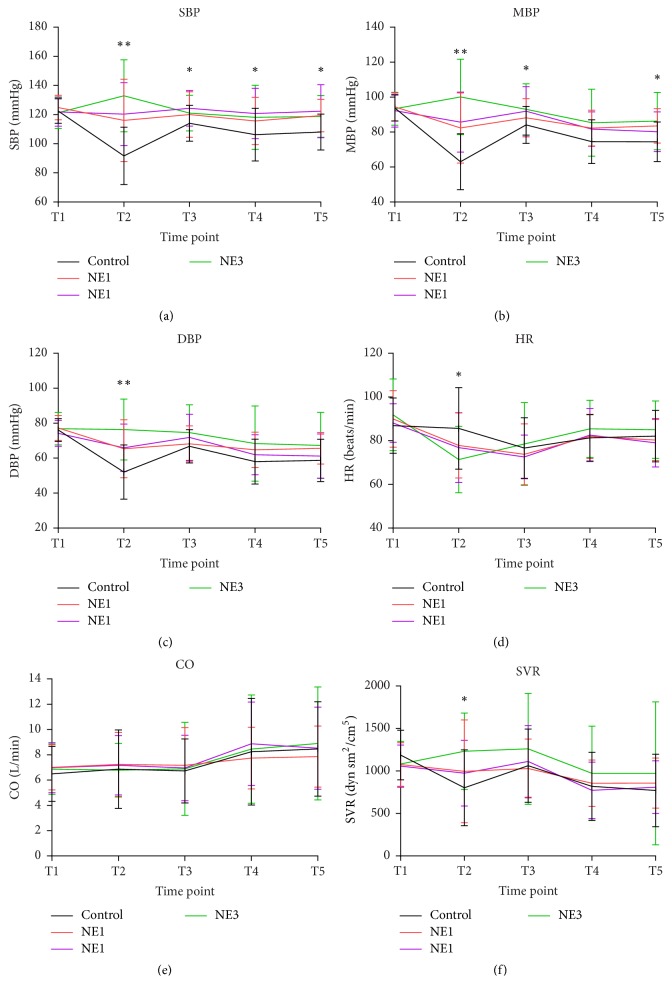
*Hemodynamic changes*. Data are shown for five timepoints: baseline (T1), the highest level of sensory block (T2), delivery (T3), oxytocin administration (T4), and end of surgery (T5). Data are presented as mean (SD). Asterisks in the upper blank indicate overall significance of each timepoint. ^*∗*^*p* < 0.05; ^*∗∗*^*p* < 0.01. Post hoc multiple comparison results are shown in supplementary Table S2.

The hemodynamic changes are shown in Figure 2. The baseline hemodynamic values were not different among these groups. Results of repeated measurement are shown in Table S1. The significance of multiple comparisons of each time point is shown in supplementary materials Table S2.

At the timepoint of the highest sensory level block (T2), SBP decreased significantly from 122.4 to 91.7 mmHg (*p* < 0.001) in the control group, which was significantly lower than in the three NE groups (*p* ≤ 0.001). SVR in the control group decreased significantly from 1188.30 to 803.167 dyn sm^2 ^cm^−5^ (*p* < 0.001). There were no significant changes in HR or CO.

At the timepoint of delivery (T3), SBP, MBP, and DBP were significantly higher (*p* < 0.001) than the values at timepoint T2 in control group. SVR increased (*p* < 0.001) and HR decreased (*p* < 0.05), while CO did not change significantly.

At the timepoint of oxytocin administration (T4), SBP had decreased from 114.03 in T3 to 106.27 mmHg (*p* < 0.05), and MBP had decreased from 84.1 to 74.53 mmHg (*p* < 0.05) in the control group. CO increased from 6.73 to 8.24 L min^−1^, and HR increased from 76.63 to 81.30 beats/min. SVR decreased significantly from 1063.03 to 819.03 dyn sm^2^ cm^−5^ in control group.

At the end of surgery (T5), the differences among groups were not significant, except for small differences in SBP and MBP between the control and norepinephrine groups.

### 3.3. Blood Gases and Apgar Scores

Maternal blood gas data and neonatal outcomes are shown in Table 4. In NE 2 and NE 3 groups, both maternal and neonatal blood glucose levels increased significantly compared to those in the control group. No significant differences in other parameters or neonatal Apgar scores among groups were detected.

### 3.4. Adverse Effects

The incidence rates of adverse effects are shown in Table 5. Though there were five participants who underwent bradycardia, most of them recovered soon without treatment. Only one patient was given atropine 0.3 mg for only once in group NE 3 and then the patient's heart rate recovered to normal without stopping or bolus norepinephrine. There were no significant differences in these adverse effects among groups.

## 4. Discussion

Our results indicated that 5 *μ*g/kg/h and 10 *μ*g/kg/h of norepinephrine significantly reduced the proportion of hypotension participants and with less incidence of hypertension and other adverse effects and would be a proper choice to prevent and treat hypotension during spinal anesthesia in cesarean sections.

The proportion of hypotension patients was significantly reduced with the use of norepinephrine infusion compared to that in the control group (overall significance *p* < 0.001). With the dosage increases, the total consumption of norepinephrine also increased; however, the proportion of hypotension was comparable between 5 *μ*g/kg/h and 10 *μ*g/kg/h (Table 3). Patients in the group treated with the highest dose of norepinephrine (15 *μ*g/kg/h) experienced more hypertension episodes. There were no significant differences among groups in terms of HR, CO, SVR, adverse effects, maternal and neonatal blood gases, or Apgar scores.

Ngan Kee et al. [10] were the first to report norepinephrine used in cesarean delivery to maintain blood pressure during spinal anesthesia in 2015. They showed that norepinephrine produced greater heart rate and cardiac output with similar antihypotension effect compared with phenylephrine by computer-controlled infusion. Vallejo [11] compared 6 *μ*g/kg/h of phenylephrine and 3 *μ*g/kg/h of norepinephrine in preventing hypotension in spinal anesthesia for elective cesarean delivery; proportion of patients who required rescue vasopressor boluses was similar between groups, so he considered norepinephrine as a alternative to phenylephrine. Ngan Kee et al. proved that manually titrated infusion of 5 *μ*g/mL of norepinephrine was effective for maintaining BP and decreasing the incidence of hypotension [13]. Our results indicated that 5–10 *μ*g/kg/h of norepinephrine infusion is suitable for maintaining blood pressure in cesarean sections which is similar to their studies.

Poterman et al. showed the antihypotension effect was equivalent with phenylephrine 100 *μ*g/min and norepinephrine 10 *μ*g/min [14]. Allen et al. [15] and Stewart et al. [7] performed phenylephrine infusion at 25, 50, 75, or 100 *μ*g/min in cesarean sections. We believe that medication according to weight is more sensible; for a 60 kg weight parturient, 2.5 *μ*g/kg/h was equal to 2.5 *μ*g/min, so we initially chose norepinephrine at 2.5 *μ*g/kg/h as a minimum dose. But in the preexperiment we found 2.5 *μ*g/kg/h could not maintain the blood pressure and a minimal dosage of 5 *μ*g/kg/h (0.08 *μ*g/kg/min) was needed to treat hypotension effectively. So we chose 5 *μ*g/kg/h as the minimum dose level and 10 and 15 *μ*g/kg/h as the middle and larger dose level. The latter two dosages may be higher than that administered in daily clinical use, because the anesthesia for our patients is usually achieved at a relatively high level (up to T5). Most importantly, we managed to maintain the hemodynamic stability for these participants under continuous monitoring.

Recently, Ilies et al. [16]. compared the continuous noninvasive arterial pressure (CNAP) device with invasive measurements in cardiovascular postsurgical intensive care patients. They concluded that the use of catecholamines including epinephrine and norepinephrine infusions did not impair the accuracy, agreement, or interchangeability of CNAP. Therefore, we used the LiDCO device to monitor hemodynamic changes and guide norepinephrine infusion.

We chose these five timepoints when hemodynamics tend to be unstable. After induction of spinal anesthesia, systemic vascular resistance decreased, while cardiac output, heart rate, and stroke volume modestly increased [8, 9]. In this study, SBP, MBP, and SVR in the control group decreased significantly after spinal anesthesia. In the norepinephrine groups, hemodynamic variables were generally stable (Figure 2). Norepinephrine has both *β*- and *α*-adrenergic activities, which might result in greater heart rate and cardiac output than phenylephrine with a lower incidence of bradycardia. However, the results from recent studies are not consistent. Ngan Kee et al. [10] reported that norepinephrine infusion during spinal anesthesia for cesarean delivery was associated with higher cardiac output than phenylephrine. Another study by Vallejo [11] found that CO was similar between the norepinephrine and phenylephrine groups.

Oxytocin is the first-line uterotonic for preventing postpartum hemorrhage. However, it may result in transient hypotension (a decrease of about 28 mmHg for 5 min) and increase heart rate and cardiac output, which contributes to unstable hemodynamics [17]. Our study also found that SBP, MBP, and SVR decreased and CO and HR increased after oxytocin administration. A previous study by Rumboll et al. suggested that phenylephrine (50 *μ*g) immediately before oxytocin injection during caesarean section did not prevent maternal hypotension and tachycardia [18]. However, in our study, it seems that the possible hemodynamic fluctuations induced by oxytocin were diminished with the use of norepinephrine.

We also found that both the maternal and neonatal blood glucose levels increased with the increasing dose of norepinephrine (Table 4). This phenomenon was found in the study by Ngan Kee et al.[10] as well as in an animal study [19]. The explanation is as follows: catecholamines regulate blood glucose [20], sometimes causing hyperglycemia [21]. Activation of *α*_1_-adrenoceptors accelerates hepatic glycogenolysis, and *α*_2_-adrenoceptors are also involved in regulating plasma glucose levels [22–24]. On the other hand, activation of *β*-adrenoceptors decreases the level of insulin. As a result, the glucose level increases in a dose-dependent manner.

It has been reported that norepinephrine infusion may cause skin necrosis [25, 26] due to its vasoconstriction effects. In this study, we monitored this adverse effect by observing the skin color. We found that the incidence of pale skin was relatively low and similar among the groups (Table 5). As a previous study showed, improved skin perfusion induced by spinal anesthesia was not counteracted by the use of norepinephrine [27], which means that norepinephrine could likely have no adverse effect on the skin perfusion in patients during spinal anesthesia.

This study has limitation that possible bias might arise as norepinephrine infusion was manually adjusted. Computer-controlled infusion might be more accurate, but it is not very widely used. Further studies are needed to strengthen our findings and investigate the effects of norepinephrine use on long-term maternal and neonatal outcomes after surgery.

## 5. Conclusions

In this study, infusion of 5–10 *μ*g/kg/h norepinephrine reduced the incidence of hypotension and better maintained hemodynamic stability during cesarean section under spinal anesthesia. In addition, no significant adverse effects on maternal and neonatal outcomes occurred.

## Figures and Tables

**Table 1:** Demographic data and surgical characteristics.

	Control (*n* = 30)	NE 1 (*n* = 29)	NE 2 (*n* = 30)	NE 3 (*n* = 28)	*p* value
Age (years)	32.9 ± 5.0	33.9 ± 3.3	33.5±3.2	32.4 ± 3.7	0.432
Weight (kg)	68.6 ± 8.1	71.3 ± 6.5	68.1 ± 9.8	69.8 ± 8.3	0.452
Height (cm)	159.2 ± 4.1	159.6 ± 4.7	159.7 ± 4.1	159.8 ± 4.1	0.960
BMI (kg/m^2^)	27.4 ± 3.5	26.7 ± 3.9	28.0 ± 3.4	27.1 ± 3.5	0.535
Gestation (weeks)	39.1 ± 1.0	39.1 ± 0.9	39.1 ± 0.7	39.0 ± 0.9	0.899
Block level (T)	4.1 ± 0.4	4.3 ± 0.6	4.2 ± 0.5	4.4 ± 0.6	0.139
SBP (baseline) (mmHg)	122.4 ± 8.4	124.9 ± 8.5	121.83 ± 9.9	121.4± 10.8	0.498
MAP (baseline) (mmHg)	93.8 ± 7.5	94.5 ± 8.1	92.3 ± 9.4	93.4 ± 9.4	0.803
DP (baseline) (mmHg)	76.2 ± 6.6	77.3 ± 7.2	74.3±7.4	76.9 ± 9.2	0.447
HR (baseline) (beats/min)	86.9 ± 12.7	90.0 ± 12.9	88.1 ± 8.8	91.9 ± 16.5	0.483
CO (baseline) (L/min)	6.6 ± 2.2	7.0 ± 1.8	6.9 ± 1.9	6.8 ± 2.0	0.844
SVR (baseline) (dyn s/cm^5^)	1188.3 ± 292.9	1075.0 ± 257.3	1058.3 ± 249.8	1082.9 ± 264.9	0.229

Data were presented as mean ± SD.

**Table 2:** Norepinephrine consumption.

	Control (*n* = 30)	NE 1 (*n* = 29)	NE 2 (*n* = 30)	NE 3 (*n* = 28)	*p* value
Time to delivery (min)	4.7 ± 1.4	5.8 ± 1.7	5.6 ± 1.9	5.5 ± 1.4	0.054
Duration of surgery (min)	41.3 ± 8.2	41.1 ± 8.7	37.6 ± 7.1	38.2 ± 8.2	0.188
Before delivery					
Infusion (*μ*g)	0.0 ± 0.0	99.8 ± 42.3^*∗∗*^	214.5 ± 69.4^*∗∗*##^	255.3 ± 113.7^*∗∗*##^	<0.001
Number of boluses (*n*)	2.0 ± 1.8	0.7 ± 1.2^*∗*^	0.6 ± 1.6^*∗*^	0.3 ± 0.7^*∗∗*^	<0.001
Total boluses (*μ*g)	19.7 ± 17.9	6.9 ± 12.0^*∗*^	6.3 ± 16.1^*∗*^	2.9 ± 6.6^*∗∗*^	<0.001
Total consumption (*µ*g)	19.7 ± 17.9	106.7 ± 44.7^*∗∗*^	220.87 ± 74.6^*∗∗*##^	258.1 ± 115.1^*∗∗*##^	<0.001

During surgery					
Infusion (*μ*g)	0.0 ± 0.0	177.9 ± 75.6^*∗∗*^	368.4 ± 134.4^*∗∗*##^	475.5 ± 241.6^*∗∗*##^	<0.001
Number of boluses (*n*)	2.3 ± 2.0	0.9 ± 1.3^*∗*^	0.7 ± 1.7^*∗*^	0.4 ± 0.8^*∗∗*^	<0.001
Total boluses (*μ*g)	23 ± 20.0	9.0 ± 13.2^*∗*^	7.3 ± 16.6^*∗*^	3.6 ± 7.8^*∗∗*^	<0.001

Total consumption (*μ*g)	23 ± 20.0	186.9 ± 79.6^*∗∗*^	375.8 ± 137.3^*∗∗*##^	479.1 ± 243.8^*∗∗*##^	<0.001

Data were presented as mean ± SD. ^*∗*^*p* < 0.05 compared with control; ^*∗∗*^*p* < 0.001 compared with control; ^##^*p* < 0.001 compared with NE 1.

**Table 3:** Hypotension and hypertension.

	Control (*n* = 30)	NE 1 (*n* = 29)	NE 2 (*n* = 30)	NE 3 (*n* = 28)	*p* value
Hypotension					
Before delivery	20 (66.7%, 48.8%–84.6%)	9^*∗*^ (31.0%, 13.1%–48.9%)	5^*∗∗*^ (16.7%, 2.5%–30.8%)	5^*∗∗*^ (17.9%, 2.7%–33%)	<0.001
After delivery	6 (20%, 4.8%–35.2%)	2 (6.9%, 0%–16.7%)	1 (3.3%, 0%–10.2%)	2 (7.1%, 0%–17.3%)	0.131
Total	26 (86.7%, 73.8%–99.6%)	11^*∗∗*^ (37.9%, 19.1%–56.7%)	6^*∗∗*^ (20.0%, 4.8%–35.2%)	7^*∗∗*^ (25.0%, 7.9%–42.1%)	<0.001

Hypertension					
Before delivery	1 (3.3%, −3.5%–10.2%)	8 (27.5%, 10.3%–44.9%)	5^##^ (16.7%, 2.5%–30.8%)	16^*∗∗*^ (57.1%, 37.6%–76.7%)	<0.001
After delivery	2 (6.7%, 2.8%–16.1%)	4 (13.8%, 0.4%–27.1%)	6 (20%, 4.8%–35.2%)	5 (17.9%, 2.7%–33%)	0.479

Total	3 (10%, −1.4%–21.4%)	12^*∗*^ (41.4%, 22.3%–60.4%)	11^#^ (36.6%, 18.4%–55%)	21^*∗∗*^ (75%, 57.9%–92.1)	<0.001

Data were presented as number (percentage, 95% confidence interval). ^*∗*^*p* < 0.007 compared with control; ^*∗∗*^*p* ≤ 0.001 compared with control; ^#^*p* < 0.007 compared with NE 3; ^##^*p* ≤ 0.001 compared with NE 3.

**Table 4:** Maternal blood gas and neonatal outcomes.

	Control (*n* = 30)	NE 1 (*n* = 29)	NE 2 (*n* = 30)	NE 3 (*n* = 28)	*p* value
Maternal blood gas					
PH	7.34 ± 0.0	7.35 ± 0.0	7.35 ± 0.0	7.36 ± 0.0	0.161
PO_2_ (mmHg)	53.4 ± 21.0	53.9 ± 22.4	48.0 ± 19.8	46.7 ± 13.9	0.381
PCO_2_ (mmHg)	41.9 ± 4.1	41.2 ± 4.8	42.6 ± 4.8	42.4 ± 4.6	0.648
Lac (mmol/L)	1.3 ± 0.5	1.2 ± 0.4	1.3 ± 0.5	1.5 ± 0.5	0.059
GLu (mmol/L)	4.5 ± 0.8	4.6 ± 0.6	4.9 ± 0.9^*∗*^	5.1 ± 0.7^*∗*#^	0.013
HCO_3_ (mmol/L)	16.1 ± 1.9	16.5 ± 2.1	15.9 ± 2.0	16.3 ± 2.0	0.680
BE	−9.7 ± 1.9	−9.0 ± 2.4	−9.7 ± 2.3	−9.2 ± 2.2	0.519

Neonatal umbilical blood gas					
PH	7.32 ± 0.0	7.33 ± 0.0	7.33 ± 0.1	7.33 ± 0.1	0.477
PO_2_ (mmHg)	23.6 ± 4.5	24.3 ± 4.2	24.7 ± 5.2	24.3 ± 6.2	0.862
PCO_2_ (mmHg)	48.6 ± 5.3	48.3 ± 6.1	50.2 ± 6.0	48.3 ± 6.0	0.545
Lac (mmol/L)	1.7 ± 0.8	1.4 ± 0.7	1.7 ± 1.1	1.8 ± 0.8	0.480
GLu (mmol/L)	3.9 ± 0.7	3.8 ± 0.6	4.2 ± 0.8^#^	4.3 ± 0.7^#^	0.047
HCO_3_ (mmol/L)	19.6 ± 2.6	20.2 ± 3.3	19.8 ± 2.5	21.0 ± 2.6	0.300
BE	−6.5 ± 2.8	−5.6 ± 3.7	−6.2 ± 3.0	−5.0 ± 2.5	0.253

Apgar 1 min	9.8 ± 0.9	10.0 ± 0.0	9.9 ± 0.3	10.0 ± 0.0	0.500
Apgar 5 min	10.0 ± 0.0	10.0 ± 0.0	10.0 ± 0.2	10.0 ± 0.0	0.412

Data were presented as mean and SD. ^*∗*^*p* < 0.05 compared with NE 0; ^#^*p* < 0.05 compared with NE 1.

**Table 5:** Adverse effects.

	Control (*n* = 30)	NE1 (*n* = 29)	NE2 (*n* = 30)	NE3 (*n* = 28)	*p* value
Shivering	8 (26.7%, 9.9%–43.5%)	4 (13.8%, 0.4%–27.1%)	4 (13.3%, 0.4%–26.2%)	7 (25.0%, 7.9%–42.1%)	0.419
Nausea	5 (16.7%, 2.5%–30.8%)	2 (6.9%, 0%–16.7%)	3 (10.0%, 0%–21.4%)	5 (17.9%, 2.7%–33%)	0.541
Pale skin	1 (3.3%, 0%–10.2%)	1 (3.4%, −3.6%–10.5%)	6 (20.0%, 4.8%–35.2%)	3 (10.7%, 0%–22.9%)	0.089
Bradycardia	0 (0.0%, 0.0%–0.0%)	1 (3.4%, 0%–10.5%)	1 (3.3%, 0%–10.2%)	3 (10.7%, 0%–22.9%)	0.232

Data were presented as number (percentage, 95% confidence interval).

## Data Availability

The data used to support the findings of this study are available from the corresponding author upon request.

## Supplementary Materials

Supplementary Material Table S1: results of repeated measurement (significance). Table S2: post hoc multiple comparison in each time point of Figure 2. Figure S1: study procedures.
