# Supplementary material for: Efficacy and Safety of Different Norepinephrine Regimens for Prevention of Spinal Hypotension in Cesarean Section: A Randomized Trial
Source: Biomed Res Int. 2018 May 23;2018:2708175. doi: 10.1155/2018/2708175 (PMC5989297; doi:10.1155/2018/2708175)
Supplement: Supplementary Material — Table S1: results of repeated measurement (significance). Table S2: post hoc multiple comparison in each time point of Figure 2. Figure S1: study procedures. [file 2708175.f1.docx]

**Supplementary tables and figures**

Table S1. Results of repeated measurement (significance)

|  | Within subject effects (time) | Between subject effects (group) | Interaction effects (time * group) |
| --- | --- | --- | --- |
| SBP | 0.003 | 0.000 | 0.000 |
| MAP | 0.000 | 0.000 | 0.000 |
| DBP | 0.000 | 0.011 | 0.005 |
| CO | 0.000 | 0.896 | 0.913 |
| SVR | 0.000 | 0.201 | 0.087 |
| HR | 0.000 | 0.612 | 0.092 |

Table S2. Post Hoc multiple comparison in each time point of fig 2

| **Group**  **Time point** | **NE1** | **NE2** | **NE3** |
| --- | --- | --- | --- |
| **T2** | **SBP****  **MBP***###**  **DBP**##** | **SBP*****  **MBP***##**  **DBP***#**  **HR***  **SVR#** | **SBP*****  **MBP*****  **DBP*****  **HR*****  **SVR***** |
| **T3** |  | **SBP****  **MBP*** | **SBP***  **MBP**** |
| **T4** |  | **SBP**** | **SBP*** |
| **T5** | **SBP****  **MBP**** | **SBP**** | **SBP***  **MBP***** |

Post Hoc multiple comparison in each time point of fig 2. Compared with control group, * p < 0.05 , ** p ≦ 0.01, *** P ≦ 0.001; compared with NE 3 group, # p < 0.05 , ## p ≦ 0.01, ### P ≦ 0.001.

**Figure S1. Study procedures**

Infusion immediately after spinal injection

80% ≤ SBP
≤ 120% of
baseline

Continuous
infusion

Hypertension
defined as SBP
> 120% of baseline

Infusion stopped

Hypotension defined as SBP <
80% of baseline and < 100 mmHg

When SBP ≤120%, infusion restarted

Bolus of 10 µg norepinephrine

Bradycardia defined as HR < 50 bpm

0.3 mg atropine
